# Mitotic Spindle Assembly in Land Plants: Molecules and Mechanisms

**DOI:** 10.3390/biology6010006

**Published:** 2017-01-25

**Authors:** Moé Yamada, Gohta Goshima

**Affiliations:** Graduate School of Science, Division of Biological Science, Nagoya University, Furo-cho, Chikusa-ku, Nagoya 464-8602, Japan; yamada.moe@a.mbox.nagoya-u.ac.jp

**Keywords:** mitosis, kinetochore, centrosome, dynein, kinesin, augmin, gamma-tubulin, *Arabidopsis thaliana*, *Physcomitrella patens*, *Haemanthus*

## Abstract

In textbooks, the mitotic spindles of plants are often described separately from those of animals. How do they differ at the molecular and mechanistic levels? In this chapter, we first outline the process of mitotic spindle assembly in animals and land plants. We next discuss the conservation of spindle assembly factors based on database searches. Searches of >100 animal spindle assembly factors showed that the genes involved in this process are well conserved in plants, with the exception of two major missing elements: centrosomal components and subunits/regulators of the cytoplasmic dynein complex. We then describe the spindle and phragmoplast assembly mechanisms based on the data obtained from robust gene loss-of-function analyses using RNA interference (RNAi) or mutant plants. Finally, we discuss future research prospects of plant spindles.

## 1. Microscopic Overview of the Spindle Assembly

**Figure 1:**
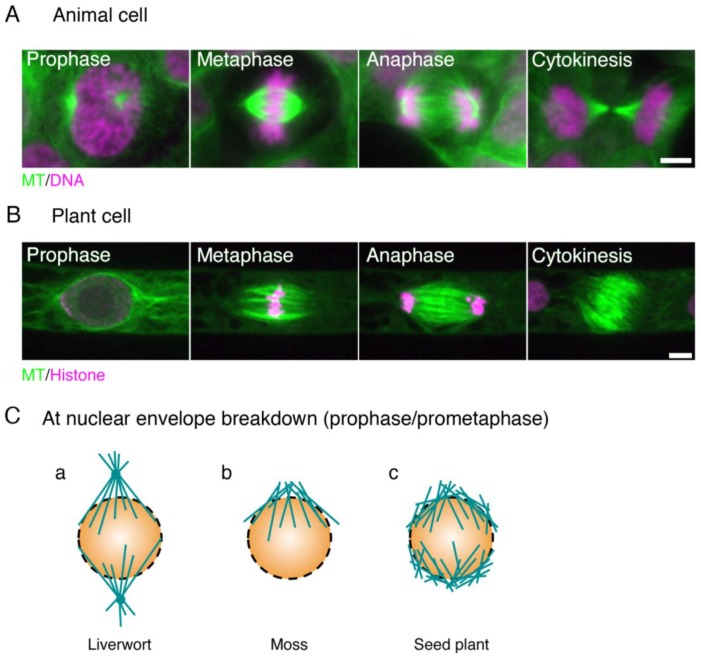
Animal and plant spindles. (**A**) Human HCT116 cells stained with anti-α-tubulin antibody (green) and DAPI (purple); (**B**) Spindles in the moss *Physcomitrella. patens* highlighted by GFP-tubulin (green) and histone H2B-RFP (purple). The two main differences between animal and plant spindles are (1) the presence of centrosomes and well-developed astral MTs in animal spindles, and (2) the morphology of the anaphase spindle (the ‘phragmoplast’ in plants). Bars, 5 µm; (**C**) distinct types of MT formation at the beginning of prometaphase (at NEBD) in liverwort, moss, and angiosperm. (**a**) In liverwort, polar organisers (POs) are assembled and act as MTOCs (microtubule-organising centres). Unlike the centrosome, however, the PO is merged into the spindle and cannot be observed as a distinct structure in metaphase [15,16]. The genes required for PO formation are unknown; (**b**) In moss protonemata, MTs are asymmetrically accumulated around the nucleus and are more abundant on the apical side [17]. An RNAi study indicated that their formation depends on γ-tubulin, but not augmin; (**c**) In most angiosperm cell types, two loosely organised MT structures known as ‘polar caps’ are detected around the nucleus [18]. γ-Tubulin is localised at this region and MTs are actively generated [19].

Mitotic spindle formation involves several key events, such as microtubule (MT) generation, bipolarity establishment, pole focusing, length control, and chromosome capture/alignment. Since the early days of spindle research, plant spindles have often been described separately from those of animal cells, perhaps owing to the apparent differences in their overall structure [1,2]. Most noticeably, land plants lack centrosomes, the dominant MT nucleating and organising centre in animal somatic cells; the metaphase spindle is generally barrel-shaped without a single focusing point at the pole (Figure 1). In the later stages of mitosis, plant cells uniquely assemble phragmoplasts that are MT arrays for cell plate material deposition. How, then, do land plant and animal spindles differ at the molecular level? In this chapter, we first outline the process of mitotic spindle assembly in animals and plants before discussing the molecular factors involved in this process.

### 1.1. Mitotic Spindle Assembly in Animals

The start of mitosis in animal somatic cells is characterised by the maturation of centrosomes during prophase. Centrosomes serve as the dominant MT generation sites as well as MT organising centres during spindle assembly [3]. After nuclear envelope breakdown (NEBD), two additional mechanisms operate to produce more MTs [4,5]. One is chromosome-mediated nucleation, in which a chromosome-associated protein activates the MT nucleation/stabilisation machinery around the chromosomes [6]. The other is MT-dependent MT nucleation, where new MTs are nucleated in a branching fashion from the existing MTs, such as those nucleated via the centrosomal or chromosomal pathway [7]. In some cell types, these three pathways act in concert, whereas in others, one or two pathways do not play major roles [8]. Regardless of their source, MTs are oriented in a bipolar manner by the action of MT-based motor proteins and through stable bipolar association with kinetochore MTs [9]. Two poles of the metaphase spindle are well focused as spindle MTs remain associated with the centrosome at the pole or crosslinked with each other by motors and MAPs [10,11]. Regulators of MT dynamics also play critical roles in spindle morphogenesis; alteration of MT dynamics affects the length and pole organisation of the metaphase spindle [12]. During prometaphase and metaphase, sister kinetochores are bioriented and attach to the plus end of MTs; completion of this process is essential for equal segregation of sister chromatids into two daughter cells. In addition, a defect in the kinetochore-MT attachment causes force imbalance in the spindle that affects spindle length. During anaphase, kinetochore MTs are depolymerised, whereas interpolar MTs elongate. In addition, MTs are de novo generated by the actions of three MT generating pathways that also operate during pre-anaphase [13]. Motor- and MAP-dependent crosslinking of those MTs at the midzone leads to the appearance of the characteristic central spindle structure during anaphase that is required for subsequent cytokinesis [14].

### 1.2. Mitotic Spindle Assembly in Seed Plants

Researchers have elucidated the mechanism of acentrosomal spindle formation in land plants through microscopic observation and have revealed the processes common to, and different from, animal somatic cells [20,21]. One of the best-characterised cell types with regard to mitotic spindle assembly is the endosperm of African blood lily *Haemanthus*. In the absence of centrosomes, abundant MTs are detected around the nuclear envelope during prophase [22]. Immunofluorescence microscopy identified MT converging centres within the MT cloud, which was consistent with the idea that they are the major MT nucleation sites at this stage [23,24]. MTs around the nucleus are gradually organised into a spindle-like structure, called the ‘prophase spindle’ (or ‘prospindle’). The prophase spindle has either a bipolar fusiform or multipolar structure [23]. After NEBD, MTs emanating from the converging centres associate with kinetochores to form kinetochore MTs [24]. MTs are also likely nucleated near the chromosome/kinetochore independent of prophase spindles during the prometaphase as an MT depolymerisation/regrowth assay detected chromosome-proximal MT formation [25]. Those MTs are then organised into an overall bipolar configuration. Electron microscopy showed that the majority of the MTs are oriented in such a way that plus ends are pointed to the chromosome/kinetochore, similar to animal spindles [26]. However, the metaphase spindle is barrel-shaped rather than fusiform, as the pole is not tightly focused at one point; multiple kinetochore and non-kinetochore MTs are converged or cross-linked locally and, thus, multiple mini-poles are observed [23]. Immunostaining of MTs also identified ‘fir tree’ structures within the spindle, in which many MTs branched off from kinetochore MTs [27]. With the start of anaphase, sister chromatids are separated and then segregated to the pole by kinetochore MT depolymerisation, analogous to animal spindles. During telophase, the phragmoplast forms and is followed by centrifugal expansion towards the cell cortex [28].

*Arabidopsis thaliana* is currently the most frequently used plant organism for genetic studies, and the mitotic spindle assembly process has been observed in several *Arabidopsis* tissues and suspension cells [19,29,30]. The tobacco BY-2 cell line is another popular system for mitosis imaging [31]. In these cells, MTs accumulate at the nuclear envelope and form prophase spindles (also called ‘polar caps’), as occurs in *Haemanthus* endosperm. Upon NEBD, MTs emanating from polar caps become a source of spindle MTs. Thus, the initial spindle assembly process in prometaphase is similar to that observed in the *Haemanthus* endosperm. The processes of metaphase, anaphase, and telophase are also analogous to those described for the endosperm. Unlike the endosperm, however, most seed-plant tissues have the preprophase band (PPB) that is a structure consisting of parallel MT arrays beneath the cell cortex that appears prior to mitosis and marks the future division plate. The PPB ensures the bipolarity of prophase spindles. While this structure is critical for division plane determination and polar cap bipolarity [32], we will not discuss this structure further in this review as they generally disappear or degenerate during the prophase and are dispensable for bipolar metaphase spindle assembly per se (see [33,34] as recent reviews on PPBs).

### 1.3. Mitotic Spindle Assembly in Bryophytes

Bryophytes have also been the subject of microscopic analysis of the mitotic spindle formation process. This process, particularly during the prophase, is somewhat different from that observed in seed plants (Figure 1C). In liverwort, prior to the appearance of prophase spindles, centrosome-like MT organising centres (MTOCs), called polar organisers (PO), appear in the cytoplasm [35,36]. The PO is similar to centrosomes in that they produce astral MTs. However, the PO is a transient structure that does not have centriole core, and during spindle formation, is no longer identified as a discrete structure. To the best of our knowledge, the roles of POs have not been experimentally demonstrated. It is, however, plausible that they function as an MT nucleation centre, as well as ensuring spindle bipolarity because they are stained well with antibodies against γ-tubulin, the major MT nucleator in eukaryotes [37,38]. In moss, conversely, PO-like structures have not been observed; instead, MTs are enriched around the nuclear envelope in prophase. These MTs emanating from the nuclear envelope represent the major source of prometaphase spindles, similar to *Haemanthus* [15,17]. In the hornwort, MTOCs are associated with plastids [15,39]. Despite the apparent differences in the earliest phase of spindle assembly, the morphology of the metaphase spindle of bryophytes is similar to that of *Haemanthus* endosperm, suggesting that a similar molecular factor is involved in the spindle assembly process during the prometaphase [15].

## 2. Conservation of Spindle Assembly Factors

The molecular factors in yeast and animal spindles have been extensively surveyed using genetics (including RNAi screening) and biochemistry (such as mass spectrometric protein identification). It is believed that most of the key factors have been identified [5,40,41,42,43]. However, since the experimental system is cumbersome and the genes are highly redundant, few genes required for spindle assembly in plants have been identified using these techniques. To characterise the putative molecular factors involved in plant spindle assembly, we performed an extensive database search (including BLAST sequence homology searching) on 131 known animal and yeast spindle factors (Table 1). As the targets, we selected *Arabidopsis* and the moss *Physcomitrella patens*, for which complete genome sequences are available [44,45], and molecular dissection of the spindle is arguably most advanced. For some genes, similar or more extensive homologue lists have previously been generated by other researchers (e.g., [46,47]); we double-checked the conservation/non-conservation of those genes with our procedure and included the references in the table.

In our search, homologues for most of the animal proteins were identified. All *Arabidopsis* genes are conserved in the moss *P. patens*; this suggests that they are likely to be found in a vast majority of land plant species. Nevertheless, our search failed to identify many components of three functional modules, namely, centrosomes, the cytoplasmic dynein complex, and kinetochores.

The homologous genes were sought as follows:

1. Animal and yeast genes required for spindle assembly were found in the literature [40,52,64,93].
2. The amino acid sequences of the *Homo sapiens* proteins were retrieved from the NCBI database. When multiple isoforms were identified, only one randomly selected isoform was used.
3. *Drosophila melanogaster* (fruit fly) and *Schizosaccharomyces. pombe* (fission yeast) homologues were sought in the NCBI ‘Homologene’ or ‘Gene’ search. When clear homologues were not identified, the BLAST search was performed.
4. Homologous genes of *Arabidopsis thaliana* and *Physcomitrella. patens* were sought using BLAST (query: human or yeast protein).
5. If no clear homologues could be identified, the databases for individual species were searched (PomBase, fly base, PHYSCObase, or TAIR). For the query, human (or, in some instances, fly) gene names or keywords (e.g., ‘centromere’, ‘kinetochore’, or ‘CENP’) were used.
6. If homologous genes were still not identified, the name was searched using Google Scholar and PubMed.
7. The sequences of plant Dsn1/Nnf1/Spc24/Ska3 and Msd1 were provided by Dr. Geert Kops (Utrecht University, The Netherlands) and Dr. Takashi Hashimoto (Nara Institute of Science and Technology, Japan), respectively.

### 2.1. Centrosome Proteins

As expected, animal centriole proteins, such as the cartwheel component Sas6, are mostly missing from the *Arabidopsis* genome. In contrast, pericentriolar proteins, like the subunits of the γ-tubulin ring complex (γ-TuRC) are more conserved. This makes sense because γ-TuRC functions at other places besides the centrosome, including the spindle MTs [15,17,58,94,95]. However, the regulators of γ-TuRC at the animal centrosome, such as the localisation factor/activator CDK5RAP2/Cnn, Plk1 kinase (polo-like kinase), and pericentrin, are largely unidentified in plants. In animals and yeasts, γ-TuRC alone lacks potent MT nucleating activity. Plants, therefore, might possess some plant-specific γ-TuRC activation factors. However, since centrosomal components are often difficult to identify by BLAST because of low sequence identity, their homologues may be present in the genome but have not yet been identified [96]. Several centriole components are found in the moss genome; these genes are likely to be required for the formation of the basal body that is used for flagella assembly in sperm [97].

### 2.2. Dynein Complex and Its Localisation Factors

Cytoplasmic dynein forms a large complex with several associated subunits and is a major MT-based motor protein in animals and fungi [98]. It moves towards the minus-end of MTs, delivers various cargoes, and generates force on the MT. In mitosis, cargoes include mitotic checkpoint proteins, chromosomes, and free cytoplasmic MTs. As previously shown [99], almost the entire dynein complex is absent from the *Arabidopsis* genome (except for the LC8 subunit that binds to other proteins such as myosin [100]). Furthermore, dynein adaptor proteins at the kinetochore or cell cortex, such as Rod, Zwilch, Spindly, or NuMA, are also missing. Thus, almost the entire dynein functional module has been lost in *Arabidopsis*. Since dynein plays various important roles in animals, plants must have developed an alternative force-generating system. One candidate is kinesin-14, which, like dynein, has minus-end-directed motility [101,102,103] and plays a partially redundant role in spindle pole organisation in animal cells [9,104]. Moss does not have cytoplasmic dynein but has axonemal dynein that is likely used for sperm motility [105]. Several dynein accessory subunits found in moss may be associated with the axonemal dynein heavy chain.

### 2.3. Kinetochore Components

Factors required for high-ordered chromosome organisation, such as condensin and cohesin complexes, and core components of the mitotic checkpoint [106] are highly conserved. However, many components of the kinetochore (the MT attachment site during mitosis) could not be identified. They might be present but could not be identified via BLAST, as kinetochore protein sequences are, in general, highly divergent among species, even within the metazoans (e.g., *Drosophila melanogaster* and humans) [107]. Current biochemical research has elucidated kinetochore subcomplexes [93]. We have identified at least one component per subcomplex in plants, with the exception of the CENP-H/I/K/M subcomplex. This suggests that other components with low sequence similarity are also present. It is also possible, however, that plants have either lost certain subunits or acquired plant-specific components. The former case is seen in *Drosophila*, in which most of the CENP components were lost during evolution. Systematic studies, such as those involving proteomics, are necessary to identify the complete set of kinetochore components in plants.

## 3. Molecular Mechanisms of Spindle Assembly in Land Plants

**Figure 2:**
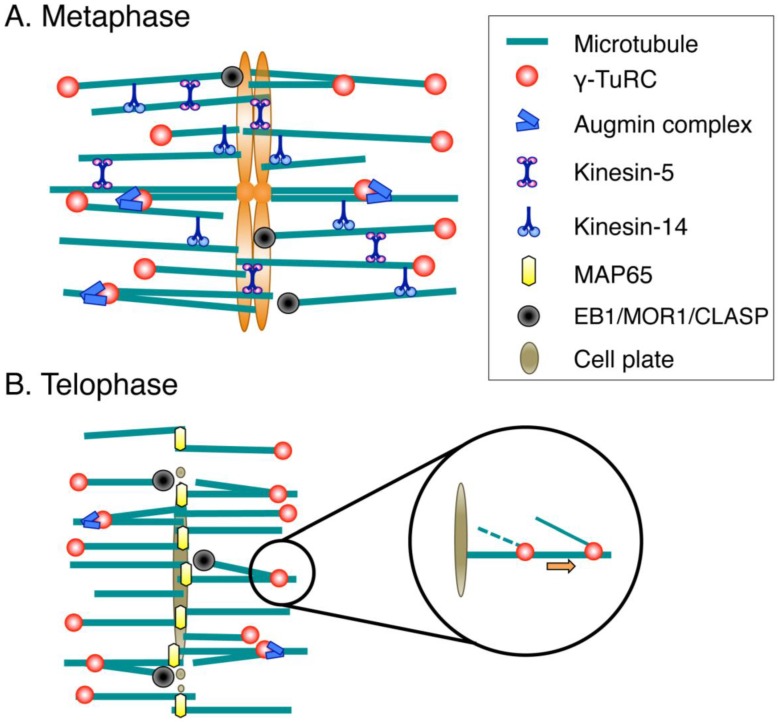
Molecular factors for spindle/phragmoplast assembly. (**A**) During prometaphase and metaphase, MTs are nucleated mainly by the γ-tubulin ring complex (γ-TuRC) and its recruitment/activation factor, the augmin complex. Multiple cross-linking proteins, including kinesin-5 and kinesin-14, shape the spindle. Spindle length is regulated by conserved MT plus-end-regulating proteins (EB1/MOR1/CLASP); (**B**) The MT-based arrays assembled after sister chromatid separation are called phragmoplasts. The central factors for MT generation in the phragmoplast are γ-TuRC and augmin, whereas MAP65 is an essential MT cross-linker that ensures phragmoplast bipolarity. MT plus ends are regulated by the same set of proteins as those acting during metaphase. Newly nucleated MTs are transported poleward via an unknown molecular mechanism.

In this section, we draw a current molecular model showing how spindles and phragmoplasts are assembled in plant cells (Figure 2). The diagram described here is based on experimental results obtained using reliable methodology such as mutant or RNAi analysis. The knowledge was derived mainly from *P. patens*, which is a system that allows rapid loss-of-function analysis and high-resolution time-lapse microscopy [108], and *Arabidopsis*, which has a rich history of mutant collection [29]. However, since genes are well conserved across land plant species, the basic mechanism could be conserved in other plant cell types. We do not include information obtained solely for animal orthologues; however, it is possible that the uncharacterised plant homologues of animal spindle proteins have identical molecular activities and functions.

### 3.1. Spindle Assembly

Genetic analyses of γ-tubulin and their associated subunits have clarified the pivotal role of the γ-TuRC in spindle MT generation [59,60,109,110]. Recently, a mechanism underlying γ-TuRC activation was also uncovered. Studies on moss and *Arabidopsis* indicated that the eight-subunit complex augmin is a key factor in increasing spindle MTs during prometaphase via γ-TuRC localisation and activation [17,65] (Figure 2A). Augmin was originally identified in *Drosophila* cells as a protein complex that drives MT-dependent MT generation by recruiting γ-tubulin onto existing spindle MTs [111]. In moss, RNAi knockdown of augmin subunits reduced MTs to ~50%, suggesting that at least half of the spindle MTs were generated via augmin-dependent, branching nucleation during prometaphase [17]. Since RNAi knockdown left behind residual augmin proteins, it is likely that 50% is an underestimate. The fir-tree structure observed in *Haemanthus* endosperm spindles might represent augmin-dependent MTs [27]. Prior to the discovery of augmin, MT-dependent MT generation was described in detail for the cortical MT arrays in tobacco and *Arabidopsis* cells [95]. Recent studies in *Arabidopsis* demonstrated that this branching nucleation is an augmin-dependent process [112]. In contrast, the origin of augmin-independent spindle MTs after augmin RNAi is unknown. It is possible that, analogous to animal cells [8], prophase MTs, and chromatin-mediated nucleation in the prometaphase (which depends on RanGTP or aurora kinase) play a role in producing these MTs.

The molecular mechanism by which plant cells achieve bipolar arrangement without centrosomes is not well understood. In animals, the key molecules in the bipolar arrangement are kinesin-5 and kinesin-12, which cross-link and slide apart anti-parallel MT overlaps in the spindle midzone [5]. This model may apply to certain plant tissues since an *Arabidopsis* mutant of kinesin-5 exhibits monopolar spindle formation in roots [113]. However, in moss protonemata, RNAi knockdown of kinesin-5 did not show monopolarisation. Moreover, GFP-tagged kinesin-5 is scarcely detected at the midzone [114]. Genes encoding kinesin-12 are amplified in plants. Therefore, it is possible that this motor redundantly plays a major role in bipolarity establishment and maintenance in some plant cell types. A comprehensive functional analysis of the kinesin-12 subfamily is required to test this hypothesis.

In animals, spindle coalescence is mediated by the partially redundant functions of kinesin-14 and cytoplasmic dynein [9,104]. Kinesin-14 has a second MT binding site in its tail domain and works as an MT cross-linker. In plants, two closely related kinesin-14 proteins, ATK1 and ATK5, have been shown to play a similar role [30,115,116,117]. When ATK5 was absent in root cells, spindles were less focused than they were in control cells [30]. However, whether the MT converging centres observed at the pole [23] are solely organised by the kinesin-14 motor remains unclear; in fly cells, this local crosslinking was dependent on an additional factor, ASPM/Asp [11,118], which is conserved, but uncharacterised, in plants.

Spindle length appears to be controlled by MT dynamics at plus ends, similar to animal cells. XMAP215/Dis1 family protein is an established MT polymerase, and the *mor1* (XMAP215/Dis1 orthologue) mutant has shorter spindles in *Arabidopsis* [67,119]. EB1 is also a critical regulator of MT plus ends, with shorter spindle formation reported for the *Arabidopsis eb1c* mutant [120]. The cytoplasmic linker-associated protein (CLASP) is an essential factor for MT polymerisation at the kinetochore in animals [121], and mutations in this gene in *Arabidopsis* resulted in significantly shorter spindles [122]. In the animal spindle, CLASP-dependent MT polymerisation and motor-dependent, poleward MT transport/sliding are coupled with minus end depolymerisation by the kinesin-13 depolymerase to maintain spindle length at the steady state (‘MT flux’; [123]). MT flux has been observed in plant spindles, suggesting that MT minus ends are also regulated by a depolymerising factor [124].

### 3.2. Phragmoplast Assembly

The phragmoplast begins to assemble upon sister chromatid separation (Figure 2B). The overall structural similarity, namely, bipolar MT array with anti-parallel MT interdigitation in the middle, have raised the notion that the phragmoplast is analogous to the central spindle or midbody in animal cells [125]. Recently reported data on MT generation further support this idea. In both structures, some MTs are constantly generated de novo in an augmin- and γ-tubulin-dependent manner [13,17]. When augmin is depleted in moss, MTs are diminished and phragmoplasts eventually disappear before they reach the cell cortex. A plausible explanation for this phenotype is that augmin utilises existing MTs, such as those carried over from metaphase, as templates for new MT nucleation. About 50% of the MTs in the central spindle of animal cells are generated in an augmin-dependent manner [13]. However, animal cells seem to have additional MT generation pathways during anaphase: at that time, hepatoma up-regulated proteins (HURP) are involved in chromosome-proximal MT generation [13]. HURP-like proteins have not been identified in the plant genome.

The key factor that maintains phragmoplast bipolarity is MAP65 (PRC1/Ase1 orthologue). This is an anti-parallel MT cross-linking protein whose activity is conserved in both yeasts and animals [126]. When three paralogous MAP65 genes are simultaneously knocked down in moss, MT bipolarity is lost and cytokinesis fails [127]. In *Arabidopsis*, bipolarity is maintained in known MAP65 mutants [128,129] but it is possible that multiple MAP65s work redundantly, and cross-linking activity persists in the mutant [130]. Other proteins, such as kinesins, might also constitute a redundant cross-linking mechanism [131].

The signalling pathway underlying phragmoplast MT regulation differs between animals and plants. In animals, the key kinases required for proper central spindle assembly are Plk1 and Aurora-B. They are concentrated at the midzone and phosphorylate multiple MT-regulating proteins including the MAP65 orthologue PRC1 [132,133]. In contrast, plants do not have Plk1. The aurora kinase constitutes a signalling pathway during cytokinesis, but the mutant exhibits a defect in orientation, but not assembly per se of the phragmoplast [134]. In plants, the MAP kinase cascade also constitutes the late mitotic signalling pathway (called the NACK-PQR pathway) [135]. One of the downstream factors in plants is also MAP65. The phosphorylation of MAP65 down-regulates its MT-bundling activity which, in turn, stimulates the progression of cytokinesis [136]. Thus, in plants, the development of MAPK signalling might have compensated for the loss of Plk1 kinase.

The phragmoplast length is regulated by MT-associated proteins; similar to metaphase spindles, shorter phragmoplasts are observed in the mutants of MOR1, EB1, and CLASP [119,120,122]. In addition, katanin-mediated severing may affect MT length in some cell types [137]. MT flux is also observed within the phragmoplast, but the molecules responsible remain to be identified [138]. 

## 4. Conclusions and Future Perspectives on Spindle Research in Plants 

At first glance, plant and animal spindles look quite different. However, the database search suggests that only a few of the mitotic elements present in animals are missing from the plant genome. Our queries using animals could not elucidate the genes that evolved uniquely in plants. Therefore, it is possible that some plant-specific genes for spindle assembly have yet to be identified. Nevertheless, most of the gene repertoire is probably common to both animals and plants. 

However, the mechanism of plant spindle assembly is formally not yet well understood. First, although gene conservation predicts that homologous proteins possess similar biochemical activity, are found in similar locations, and execute similar functions, each of these *must* be tested experimentally. Indeed, recent ‘repeat’ experiments using the plant orthologues of well-characterised animal genes have revealed unexpected functions, such as the role of kinesin-5 in chromosome alignment or cytokinesis [114] and a γ-TuRC-interacting protein in centromere integrity [61]. Regarding localisation dynamics, a comprehensive study in moss showed that 42 out of 43 mitotic kinesins were localised at a site not observed in animal studies [114]. Until recently, ‘repeat’ experiments intended to confirm animal study results were very time-consuming due to the lack of a model cell system for rapid investigation. The recent development of quick and robust loss-of-function tools such as conditional RNAi and CRISPR/Cas9-based genome editing technology in moss and liverwort, as well as advances in live microscopy, have provided an opportunity to delve into *putative* spindle assembly factors in plants [17,139,140,141,142].

Second, it is not yet known how plants compensate for the lack of two major components, centrosomes and dynein. In animal and yeast, these two components are critical for spindle positioning, which is a crucial process in determining the cell division axis and symmetry/asymmetry [143]. It has been shown that genetic perturbation of the PPB causes division axis abnormalities in seed plants [33,34]. Yet little is known about the molecular mechanism underlying the PPB-dependent determination of the spindle axis. It is remarkable that plants evolved a unique PPB-based mechanism to substitute for centrosome function [144]. The mechanism by which plant spindles are oriented in the proper direction remains a fascinating, and as yet unanswered, question.

## Figures and Tables

**Table 1:** List of homologues of spindle assembly factors.

	Generic Name	*H. sapiens*			*D. melanogaster*			*S. pombe*			*P. patens*			A. thaliana (* identified with BLAST)			*A. thaliana* Gene Acession #	References
Kinase/phosphatase/signalling	Cdk1		+			+			+							*	AT3G48750	
	Aurora kinase		+			+			+			+			+	*	AT4G32830 etc.	[48]
	Plk1		+			+			+									[49]
	Haspin		+			+			+			+			+	*	AT1G09450	
	Ran		+			+			+			+			+	*	AT5G20010 etc.	
	RCC1		+			+			+			+			+	*	AT5G63860 etc.	
	RanGAP		+			+			+			+			+	*	AT3G63130, AT5G19320	
	PP2A		+			+			+			+			+	*	AT1G69960 etc.	[50]
	Endosulfine		+			+			+			+			+		AT1G69510	[51]
	PP1		+			+			+			+			+		AT2G29400	[50]
	PP6		+			+			+			+			+	*	AT3G19980	
Centriole	Plk4		+			+												[52]
	Sas4		+			+												
	Sas5/Ana2/STIL		+			+												
	Sas6		+			+												[52]
	Spd2/CEP192		+			+												
	Ana1/CEP295		+			+												
	Ana3/Rotatin		+			+												
Motor/MAPs	Kin4/chromokinesin		+			+						+			+	*	AT5G60930 etc.	[53,54]
	Kin5		+			+			+			+			+	*	AT2G28620 etc.	
	Kin6		+			+												
	Kin7/CENP-E		+			+						+			+	*	AT3G10180 etc.	
	Kin8		+			+			+			+			+	*	AT1G18550, AT3G49650	
	Kin12/KIF15		+									+			+	*	AT3G19050 etc.	
	Kin13		+			+						+			+	*	AT3G16060, AT3G16630	
	Kin14		+			+			+			+			+	*	AT4G21270 etc.	
	DHC		+			+			+									[55]
	DIC		+			+			+									
	DLC (LC8)		+			+			+			+			+	*	AT4G15930 etc.	
	Dynactin p50		+			+			+									
	Dynactin p150		+			+			+									
	Dynactin ARP1		+			+			+									[56]
	PRC1/MAP65/Ase1		+			+			+			+			+	*	AT3G60840 etc.	
	Katanin (p60)															*	AT1G80350	
	HURP		+			+												
	TACC		+			+			+									
	TPX2		+			+						+			+	*	AT1G03780 etc.	[57]
Nucleation	γ-Tubulin		+			+			+			+			+	*	AT3G61650, AT5G05620	[58]
	GCP2/3		+			+			+			+			+	*	AT5G17410, AT5G06680	[46]
	GCP4/5/6		+			+			+			+			+	*	At3g53760 etc.	[46,59]
	NEDD1		+			+						+			+	*	AT5G05970	[60]
	Mzt1		+			+			+			+			+	*	AT1G73790, AT4G09550	[61,62,63]
	Mzt2		+															[64]
	Augmin (8 subunits)		+			+						+			+	*	At5g40740 etc.	[17,46,65]
	Pericentrin/D-plp		+			+												[64]
	AKAP9		+															[64,66]
	SPC110/Pcp1								+									[64]
	CDK5RAP2/Cnn		+			+			+									
	Myomegalin		+															
Microtubule plus end	ch-TOG/XMAP215		+			+			+			+			+	*	AT2G35630	[67]
	EB1		+			+			+			+			+	*	AT5G62500 etc.	
	SLAIN/Sentin		+			+												
	CLIP170		+			+			+									
	CLASP		+			+			+			+			+	*	AT2G20190	
	SKAP		+															
	Astrin		+															
Microtubule minus end	CAMSAP		+			+												[68]
	Msd1/SSX2IP		+						+							*	AT5G57410 etc.	[69]
	ASPM		+			+						+			+	*	AT4G21820	
	CaM		+			+			+			+			+	*	AT2G27030 etc.	
	NuMA		+			+												
	Microspherule		+			+						+			+	*	AT3G54350 etc.	
Chromosome	CAP-D2		+			+			+			+			+	*	AT3G57060	[70]
	SMC2		+			+			+			+			+	*	AT3G47460, AT5G62410	[70,71]
	CAP-H		+			+			+			+			+	*	AT2G32590	[70,72]
	SMC4		+			+			+			+			+	*	AT5G48600	[70,71,72,73]
	CAP-G		+			+			+			+			+	*	AT5G37630	[70]
	Topo II		+			+			+			+			+	*	AT3G23890	
	Rad21		+			+			+			+			+	*	AT5G16270 etc.	[74,75]
	SCC3		+			+			+			+			+	*	AT2G47980	[75]
	SMC1		+			+			+			+			+	*	AT3G54670	
	SMC3		+			+			+			+			+	*	AT2G27170	
	SCC2		+			+			+			+			+	*	AT5G15540	[76]
	SCC4		+			+			+			+			+	*	AT5G51340	
	Eco1		+			+			+			+			+	*	AT4G31400	[77]
	Sororin		+			+												[78]
	Wapl		+			+			+			+			+	*	AT1G11060	
	PDS5		+			+			+			+			+	*	AT5G47690 etc.	
Kinetochore/centromere	HP1		+			+			+			+			+	*	AT5G17690	
	Sgo1		+			+			+			+			+		AT3G10440, AT5G04320	[79]
	Borealin		+			+			+							*	AT4g39630	
	INCENP		+			+			+			+			+		AT5g55820	[80]
	Survivin		+			+			+									
	CENP-B		+						+									
	Mis18		+						+									
	Mis18BP1		+						+			+			+		At5g02520	[81]
	HJURP		+						+									
	Cal1					+												
	CENP-A		+			+			+			+			+	*	AT1G01370	[82]
	CENP-C		+			+			+			+			+	*	AT1G15660	[83]
	CENP-S		+						+			+			+	*	AT5G50930	[84]
	CENP-X		+						+			+			+		AT1G78790	[85]
	CENP-T		+						+									
	CENP-W		+						+									
	CENP-L		+						+									
	CENP-N		+						+									
	CENP-H		+						+									
	CENP-I		+						;									
	CENP-K		+						+									
	CENP-M		+															
	CENP-O		+						+			+			+	*	AT5G10710	
	CENP-P		+						+									
	CENP-Q		+						+									
	CENP-U		+						+									
	CENP-R		+															
	Mis12		+			+			+			+			+		AT5G35520	[86]
	Dsn1/Mis13		+						+						+		AT3G27520	
	Nnf1		+			+			+						+		AT4G19350	
	Nsl1/Mis14		+			+			+									
	KNL1		+			+			+								AT2G04235	[87]
	Ndc80		+			+			+			+			+	*	AT3G54630	
	Nuf2		+			+			+			+			+	*	AT1G61000	
	Spc24		+			?			+			+			+		AT3G08880, AT5G01570	
	Spc25		+			+			+			+			+	*	AT3G48210	
	Ska1											+			+	*	AT3G60660	
	Ska2																AT2G24970	
	Ska3		+									+			+		AT5G06590	
	Dam1								+									
	CENP-F		+															
Spindle assembly checkpoint (SAC)	Mad1		+			+			+			+			+	*	AT5G49880	
	Mad2		+			+			+			+			+	*	AT3G25980	[88]
	Mad3 /BubR1		+			+			+			+			+	*	AT2G33560, AT5G05510	[88,89]
	Bub1		+			+			+			+			+	*	AT2G20635	
	Bub3		+			+			+			+			+	*	AT3G19590, AT1G49910	
	Mps1		+			+			+			+			+	*	AT1G77720	[90]
	Tpr		+			+			+			+			+	*	AT1G79280	[91]
	Cdc20		+			+			+			+			+	*	AT4G33270 etc.	
	Spindly		+			+												
	Rod		+			+												
	Zwilch		+			+												
	Zw10		+			+						+			+	*	AT2G32900	[92]

Black boxes indicate that homologous genes are present. Boxes are left blank if no clear homologues are present. The *Arabidopsis* genes identified with the BLAST search are marked with asterisks (*). Gene accession numbers for *Arabidopsis* genes were presented; however, just one or two numbers were provided when more homologues were identified or for protein complexes (augmin and GCPs). The identities of other subunits are found in the references presented in a separate column.
